# The Evolution of the Malaria Clinic: The Cornerstone of Malaria Elimination in Thailand

**DOI:** 10.3390/tropicalmed4040143

**Published:** 2019-12-13

**Authors:** Prayuth Sudathip, Suravadee Kitchakarn, Krongthong Thimasarn, Deyer Gopinath, Tinzar Naing, Omar Sajjad, Sumetha Hengprasert

**Affiliations:** 1 Department of Vector Borne Diseases, Department of Disease Control, Ministry of Public Health, Nonthaburi 11000, Thailand; p.sudathip@gmail.com (P.S.); kitchakarn@hotmail.com (S.K.); 2 Independent Senior Malaria Consultant, Bangkok 10220, Thailand; krongtho95@yahoo.com; 3 World Health Organization, Country office Thailand, Nonthaburi 11000, Thailand; 4 Principal Recipient Office for Global Fund to fight AIDS, Tuberculosis and Malaria, Department of Disease Control, Ministry of Public Health, Nonthaburi 11000, Thailand; tinzar.nd@gmail.com; 5 Intern, World Health Organization, Country office Thailand, Nonthaburi 11000, Thailand; Omar.Sajjad@ucsf.edu; 6 Independent Malaria Consultant, Nonthaburi 11000, Thailand; sumethah@yahoo.com

**Keywords:** malaria control, malaria elimination, Thailand, malaria clinics

## Abstract

**Background:** Malaria Clinics (MCs) have served communities in Thailand since 1965 and are still playing a critical role in providing early diagnosis and effective treatment of malaria. **Methods:** We reviewed six decades of published manuscripts, articles, strategies, and plans regarding MC operations in Thailand;,and analyzed national program surveillance data in both malaria control and malaria elimination phases. **Results:** MCs accounted for 39.8% of malaria tests and 54.8% of positive cases by the end of the 1980s. The highest number of MCs established was 544 in 1997. MCs contributed to 6.7% of all tests and 30% of all positive cases over the 2015–2017 period. Between 2017 and June 2019, during the malaria elimination phase, MCs continued to test an average of 67% of all persons tested for malaria, and confirmed 38.3% of all positive cases detected in the country. **Conclusions:** Testing and positive rates of MCs are on a gradual decline as the overall burden of malaria declines annually, which may reflect decreasing transmission intensity. Although the number of MCs in the last three years has been stable (*n* = 240), the attrition of MC staff poses a real challenge to the longevity of MCs in the absence of a human resource plan to support the elimination phase. It is necessary to identify and support capacity gaps and needs as MCs are absorbed into an integrated and decentralized program, while ensuring that the Division of Vector Borne Diseases (DVBD) maintains its necessary technical and advisory role.

## 1. Introduction

### 1.1. Brief History of Anti-Malaria Operations in Thailand

The first recorded anti-malaria operation commenced in 1930 in northern Thailand, where efforts were limited to administering quinine and reporting annual malaria mortality [1]. In 1932, the first “Shivering Fever Unit” was established in Chiang Mai province to clinically investigate and treat malaria patients with quinine free of cost, and to educate the population on how to avoid mosquito bites. In 1944, the centrally led Malaria Control Unit was upgraded to the Malaria Division within the Ministry of Public Health (MoPH) and was comprised of five zone offices in different regions of Thailand. Each zone had five malaria units and totaled 25 malaria units in 1945.

In 1949, malaria was the leading cause of mortality in Thailand with over 38,000 deaths, a rate of 2015 per 100,000 population [1]. **A 2-Year Malaria Control Demonstration Project (1949–1951)** was supported by the World Health Organization (WHO) and UNICEF as a pilot project in Chiang Mai province using indoor residual spraying (IRS) with dichlorodiphenyltrichloroethane (DDT). The results were encouraging, leading the Thai government (in collaboration with the WHO and the United States government) to develop a country-wide Malaria Control Program (**MCP) from 1951–1957**, covering 61 provinces within ten years [2,3]. The MCP adopted, evaluated, and revised various malaria control strategies from existing vector control methods, such as the countrywide spraying of DDT, along with anti-malaria drug distribution to at-risk populations concentrated in forested areas [4]. In addition to these strategies, malaria clinics (MCs) established since the 1960s onwards have played a critical role for the MCP in providing early diagnosis and treatment for malaria. As a result of these combined strategies, the malaria death rate declined from 183.1 per 100,000 population in 1950, to 43.0 per 100,000 population in 1957, and further to 22.8 per 100,000 population in 1963 [5].

In 1958, the Malaria Division began reorienting its MCP toward a **Malaria Eradication Program (MEP; 1965–1970)** in accordance with WHO guidance. The MEP was initiated in 1965. In 1966, there were 40 malaria laboratories performing malaria microscopy at the Malaria Division headquarters, regional offices, provincial malaria zone offices, and district malaria sector offices.

The malaria eradication effort ceased in 1971, and the government developed the **MCP Plan (1971–1976)** with a focus on remote and forested areas. This program aimed for control in areas of medium and high receptivity and eradication in areas of low receptivity, where malaria services were already partially integrated within the general health services. Receptivity being defined as presence/absence of *Anopheles* species.

Increasing malaria rates in the late 1970s ushered in an **MCP Reformation (1977–1996)**, which aimed to provide a comprehensive control program to all at-risk populations in Thailand. During the 1980s, the MCP faced several operational and financial constraints. The massive influx of refugees from Cambodia and the emergence and rapid spread of *Plasmodium falciparum* (*P. falciparum)* resistance to chloroquine and sulfadoxine/pyrimethamine resulted in malaria epidemics along the Thai–Cambodia border [6]. Elsewhere in the country, malaria morbidity and mortality declined, with some fluctuations due to small-scale outbreaks. Malaria was highly prevalent in forested, mountainous areas along the borders (especially the western and southeastern borders) and the southern peninsula, but the central region (plains areas) was largely left malaria-free [7].

**Figure 1:**
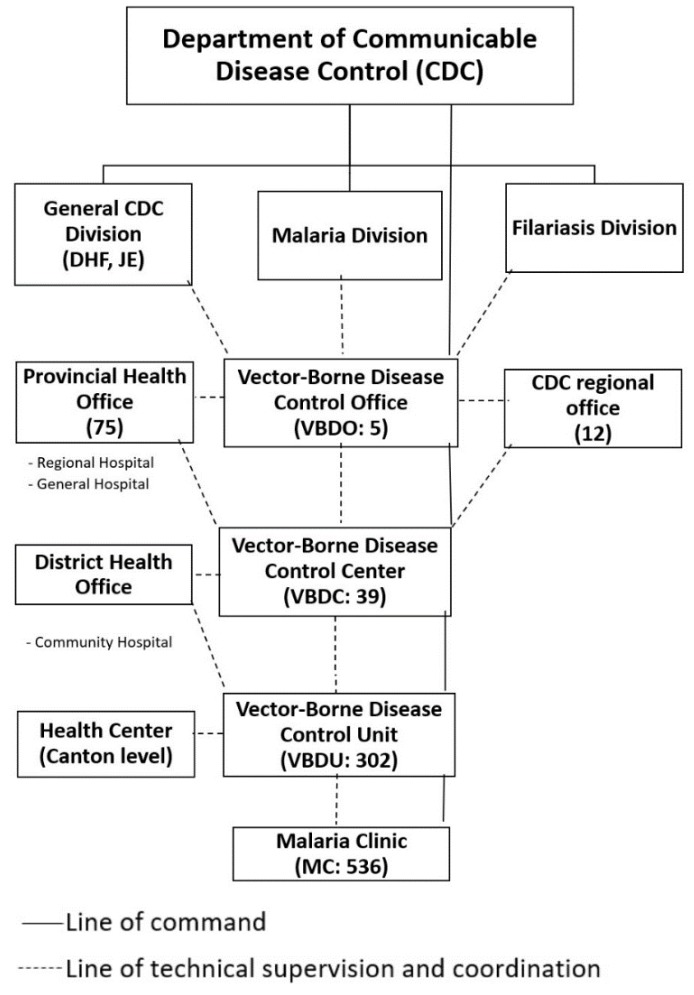
Organization chart of the Vector Borne Disease Control Programme, Thailand in 1999.

In 1995, following the adoption of the Global Malaria Control Strategy, the MCP under the **Eighth Five-Year National Health Development Plan (1997–2001)** aimed to reduce malaria morbidity and mortality with special attention on 30 border provinces. The MCP remained organizationally separate and vertical, although some activities, such as case detection, had been partially integrated into the general health services, especially in low malaria areas. Vector control activities and active and passive case finding at specialized MCs remained under the responsibility of the MCP structure [3]. **Reorganization of the MCP and integration into general health services** commenced in 1996 (Figure 1) in response to financial constraints, downsizing of government institutes, and the steady decline of malaria over the previous decade. The MCP merged with the Filariasis and Dengue Hemorrhagic Fever Control Programs at the regional and provincial levels.

**Figure 2:**
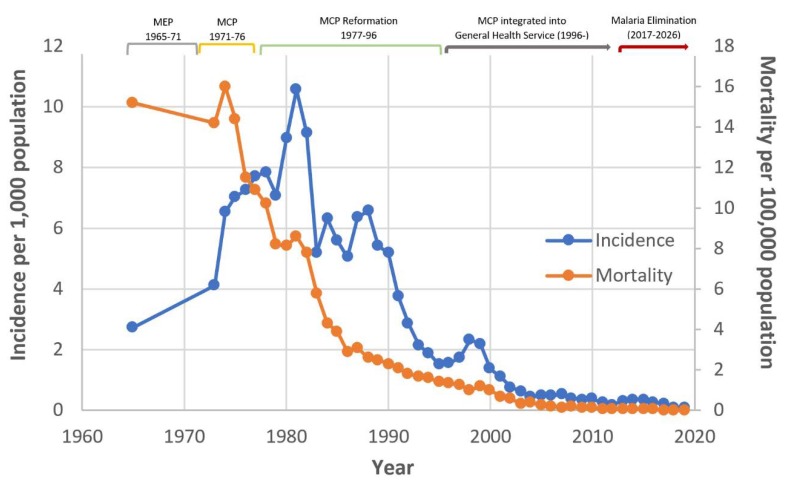
Malaria incidence per 1000 population and malaria mortality per 100,000 population in Thailand over phases of program orientation between 1965 and 2019*. * Data until June 2019.

In the new millennium, total malaria cases and deaths continued to decline. The Malaria Division became the National Vector Borne Disease Division in 2002; the integration of regional and provincial level vector borne disease (VBD) structures with other communicable disease programs occurred simultaneously. The malaria program remained semi-vertical within the Department of Communicable Disease Control (now the Department of Disease Control). Thailand saw a continuous decline in malaria burden from 64,957 reported cases in 2010 to 17,153 in 2016 and a morbidity rate of 0.38/1000 population, with much of the malaria transmission concentrated along international borders. This success paved the way for the Ministry of Public Health to develop a **Malaria Elimination Strategic Plan for Thailand 2017–2026** with the vision that Thailand will be malaria-free by 2024. To drive the Strategic Plan and monitor implementation progress, a different mechanism was required at the national level, i.e., the Committee on Sustainable Development Goals. Therefore, the Steering Committee on Malaria Elimination and the Administrative Committee on Malaria Elimination were established. At the provincial level, the Office of Public Health Inspectors, the Office of the Permanent Secretary, and the Department of Disease Control are responsible for transferring the policy, guidelines, and interventions through the Provincial Communicable Disease Committees, Communicable Disease Control Units (CDCU), the health facility units of both public and private sectors, and civil society organizations to implement the malaria elimination plan according to the local context of each area. [8]. Thailand envisions the elimination of malaria by 2024 and aims to achieve this in more than 95% of districts by 2021, with all districts malaria-free by 2024. As Thailand has re-orientated its malaria control program to an elimination program, malaria stratification has been reclassified based on the district as the lowest administrative unit for elimination and updates on village-level malaria risk are issued annually. [9]. Thailand has made rapid progress in targeting and reducing the number of active foci (villages) of transmission in the country from 1542 in 2015 to 415 in early 2018. The total number of confirmed malaria cases was reduced from 17,153 in 2016 to 13,974 in 2017. Remaining active foci are primarily located in three border areas: In the west with Burma; in the east with Cambodia, where high population mobility is associated with importation of malaria parasites and complicates surveillance; and in the south with Malaysia, where civil unrest inhibits service delivery [10]. Overall trends in malaria incidence and mortality over 1965–2019 (until June) are summarized in Figure 2.

### 1.2. House Visitor: The Role in 1960–1979 for Malaria Diagnosis–Treatment

Prior to the establishment of MCs, patients had to wait for house visitors (HVs) to visit households within their assigned areas at least once a month [1]. Since 1965, HVs were contracted as permanent employees and the recruitment criteria were at least a secondary school education and one month of pre-service training. The training included knowledge of malaria, preparing blood films, pre-staining slides for microscopy, and health education. Usually, HVs would ask five screening questions about each member of the household, such as presence of fever and history of visiting malaria endemic areas. A blood film was prepared for those who replied “yes” to at least one question. For symptomatic individuals, “presumptive treatment” consisting of chloroquine and pyrimethamine (for adults) was given to control the spread of malaria infection and reduce mortality. The HV would return to the Malaria Zone offices in the last week of the month to send the blood slides to the laboratory, submit blood record forms, and prepare materials for the next round. The blood slides were examined at the Malaria Zone Office; if found to be positive for malaria, the HVs would return the following month to give “radical treatment”. The average turnaround time from slide collection to radical treatment was around one month, but sometimes longer in laboratories with a high volume of slides [11]. Besides the routine HV activities, squad chiefs were appointed as the senior HVs in a given area to coordinate and supervise the HVs. HV squad chiefs intermittently collected blood films and sent these to the provincial-level Malaria Zone Office laboratory. Similarly, the chiefs of district-level Malaria Sector Offices were also tasked to investigate and treat cases. MCs were initially staffed by HVs, who were formally re-designated as “Malaria Clinic Workers” in 1979 and stationed at Malaria Sector Offices [12].

### 1.3. Human Resources in the MC

There are three main categories of government-employed staff in Thailand. First, Civil servants, such as doctors, nurses, technical staff, administration staff, heads of the vector borne disease control center (VBDC), vector borne disease control unit (VBDU), etc., are eligible for training opportunities and promotion to higher positions. Second, there are permanent employees, like HVs, senior HVs, chief of malaria spray teams, drivers, laboratory staff at the VBDC, data entry staff, entomology field workers, etc. Job descriptions are generally fixed with very little change over the years. Training opportunities have been limited, and promotion requires passing relevant examinations. However, this category offers better job security than temporary employees, who are recruited for specific periods and tasks like project staff, spray team members, etc.

Over the years, HVs, senior HVs, and some chiefs of malaria spray teams who fulfilled the criteria were employed as MC workers and remained as “permanent employees”. Although they remained in the same position (of HV) with the same salary scales, many HVs opted to work as MC workers and received the local community’s support and respect [13] personal records.

Prior to the 1980s, when more civil servant positions were created, MC workers had promotion opportunities and could pass the required examinations easily, having more knowledge of malaria and public health than most other applicants. This saw many of the House Visitors and MC workers become heads of the VBDC and VBDU. However, from 1980 onward, this practice stopped and VBDU staff were recruited and trained as other staff of the Provincial Health Office (PHO) (personal records).

In the 1990s, the government imposed a new rule on downsizing government staff recruitment. Recruitment for vacant permanent employee positions was stopped. However, as MC workers were still relatively young, this had little effect till the 2000s and onward, when many MCs were closed largely due to MC worker retirement and attrition. In addition, unlike civil servant positions, MC workers could not be reassigned to a new district or province where they were needed most, as they were not entitled to house rent and other subsidies (personal interviews and records).

## 2. Materials and Methods 

The objective of this study was twofold. Firstly, to document the role of MCs and analyze quantitatively the impact in providing early diagnosis and effective treatment of malaria; and secondly, to assess the relevance of MCs in the context of malaria elimination in Thailand. We performed a literature and document review regarding MCP and MCs from published articles, unpublished reports and data, annual reports (ARs) of the Malaria Division, Department of Communicable Disease Control, Ministry of Public Health, Thailand from 1975 to 2018, and personal records of the Division of Vector Borne Diseases (DVBD) senior staff members. Aside from journal published articles, all records were in Thai and required the translation of S.H on relevant sections. We discussed the future factors most affecting the work of the MCP and MCs with senior staff members of the DVBD. To protect the confidentiality of the interviewers, their opinions were given without any links to their names or organizations. Personal records were obtained from two of the senior authors. All information was reviewed and analyzed. Since this article did not involve personal identifiable data and used only secondary data, an information sheet and consent forms were exempted.

Data for malaria incidence and mortality, number of MCs, percentages of slides examined, and positive cases detected at MCs were obtained from the DVBD, Department of Disease Control, Ministry of Public Health, Thailand. Malaria incidence and mortality were each plotted against the number of MCs from 1965 to June 2019. Number of slides examined and number of positive cases detected at MCs were each analyzed in three-year increments. The years 1965–1974 were omitted because of missing data from MCs in some years. Tables 1 and 4–7 were created using the Self Analysis tool on the Thailand GMS Malaria website: http://thailand.gmsmalaria.org/. Cases were filtered by year, foci, case classification, nationality, and province. To find cases from MCs specifically, cases were further filtered by health facility. All *p*-values were calculated via univariate chi-squared tests in the R statistical program (version 3.5.2; Foundation for Statistical Computing, Vienna, Austria, 2013).

## 3. Results

### 3.1. Malaria Clinics (MCs): The Early Years in 1960–1980

Although the first documented MC was established in Pakchong District, Nakorn Ratchasima province in 1959, the precise number of MCs that were subsequently established was undocumented until 1975, when 14 MCs that were established at Malaria Sector Offices were documented. In the initial years from 1960–1980, the term “malaria clinic” was not officially used. Malaria staff referred to it as a passive case detection (PCD) post and added “office for free and prompt treatment of malaria” on the signage outside the facility. The term “malaria clinic” was discussed and introduced in a Regional WHO malaria meeting in 1977, but was not officially implemented until the early 1980s, when United States Agency for International Development (USAID) provided financial support for expansion of the MC network [1,3].

MCs were initially staffed by HVs. In 1975, a formal 8-week training course for malaria HVs stationed at MCs was introduced to include the basic principles of malaria transmission, English terminologies, and training on microscope usage for the examination of blood films. The prerequisite educational qualification for MC workers was raised to a minimum level of 13th grade (equivalent of completion of high school). Primary duties included blood taking, staining blood films with Giemsa, microscopic examination of blood slides, face-to-face health education, and record form submission. Additional duties included conducting case investigations under the supervision of the Malaria Sector chief and in some MCs with low workload, microscopic examination of blood slides from Active Case Detection (ACD) activities. The Malaria Division at the central level mandated that MC workers should manage no more than 30 patients per day and should not conduct more than 60 microscopic slide examinations per day (personal interviews) [1,3,4]. Following completion of this training, HVs were renamed as “house visitors for diagnosis–treatment”. This designation was used until 1979, when the staff position was re-profiled as “Malaria Clinic Worker” and was officially stationed at Malaria Sector Offices. This was the beginning of the setup of MCs within Malaria Sector Offices, as well as the expansion of malaria clinics (MCs) outside of the sector offices (stand-alone malaria clinics) [1,3]. MCs were equipped with light microscopes and a minimal set of laboratory supplies. The patient waiting time between diagnosis and treatment was reduced to 30–40 min as compared to a month during the house visiting phase prior to 1975. Patient satisfaction with this service further established the popularity of MCs [14].

**Figure 3:**
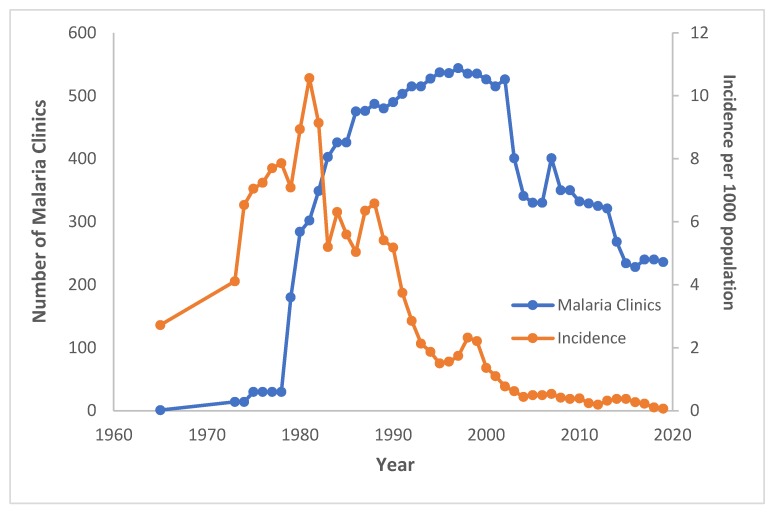
Number of malaria clinics (MCs) and malaria incidence per 1000 population in Thailand between 1965 and 2019.

**Figure 4:**
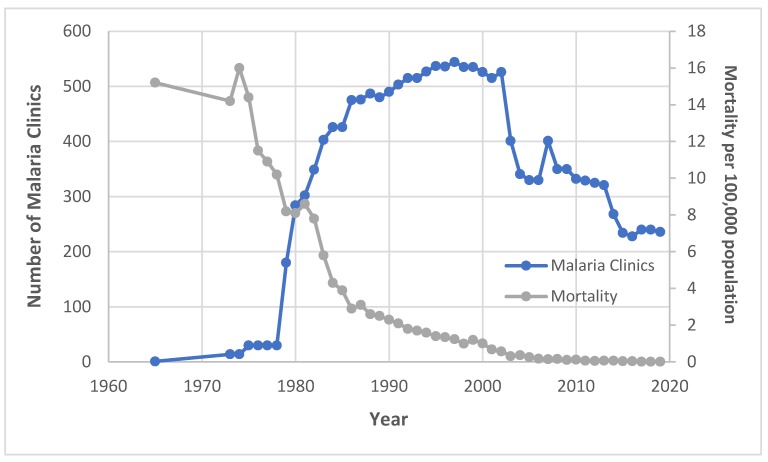
Number of MCs and malaria mortality per 100,000 population in Thailand between 1965 and 2019.

**Figure 5:**
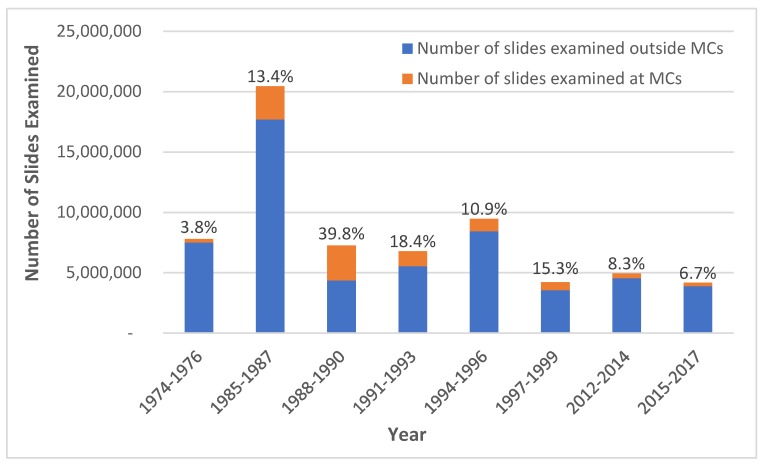
Percentage of slides examined by MCs for malaria between 1974 and 2017.

During this early period of few MCs, malaria incidence more than tripled (2.72 to 8.94 cases per 1000 population) over the fifteen-year period between 1965, when incidence data were first made available, and 1980 (Figure 3). Meanwhile, malaria mortality dropped by half (16 to 8.1 deaths per 100,000 population) between 1974 and 1980, a trend that predates MC expansion by roughly four years (Figure 4). From 1974 to 1976, MCs were responsible for examining only 3.8% (*n* = 296,344) of all slides tested for malaria in Thailand (Figure 5) [5,11].

### 3.2. Malaria Clinics (MCs): The Expansio in: 1980–2000 

In 1978, there were a total of 30 MCs in Thailand. By 1980, a USAID project for malaria control supported an expansion to 284 malaria clinics (AR, 1980) (Figure 3 and Figure 4). A national MC policy was established by the Malaria Division in the same year, which provided a clearer typology of MCs based on their location and function. The first type of MC was a “Fixed MC”, located in Malaria Sector Offices (equivalent to district VBDUs at present), outside malaria offices as stand-alone MCs, or within public health facilities like health centers. Fixed MCs were mainly along main routes/entry to transmission areas, check points, or insecure areas unsuitable for operations such as armed-conflict areas in the south of Thailand. The second type of MC was a “Mobile MC”, which was a service offered by sending a team of two to three malaria staff to hard-to-reach, epidemic-prone, or high-incidence areas [15]. The sites for mobile MCs were places in which either local communities or villagers from remote, high endemic areas usually gathered intermittently. There were two kinds of mobile MCs: (1) Mobile malaria clinics (MMCs), which were ad-hoc/non-scheduled at unplanned sites; and (2) fixed schedule mobile clinics (FSMCs), which were planned in time, duration, and location with participation from the community. During malaria outbreaks, FSMCs could be set up during Sunday market or festivals and would be terminated once malaria prevalence was reduced [16].

From 1980 onwards, more MCs were established, both within Malaria Sector Offices and as stand-alone MCs. Between 1980 to 1988, in response to major outbreaks, especially in border regions to Cambodia, the government increased its funding for malaria control. It subsequently established more malaria clinics, strengthened program infrastructure, and contributed significantly to a rapid decline in cases from an annual malaria incidence of 10.6 per 1000 population in 1981 (450,000 cases) to 6.8 per 1000 population by 1988.

**Figure 6:**
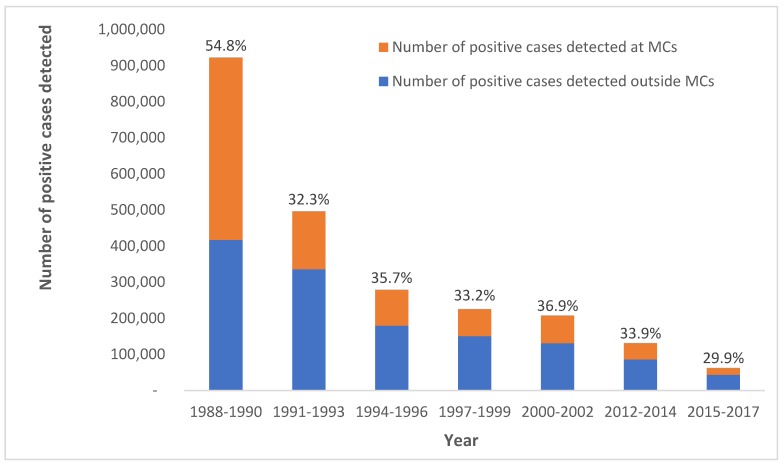
Percentage of positive cases of malaria detected by MCs between 1988 and 2017.

There was a total of 302 MCs by the end of 1981. By 1985, MCs accounted for more than 60% of all malaria cases detected (Figure 6). Although cases dropped as low as 198,000 cases in 1991, *Plasmodium vivax (P. vivax)* outbreaks in coffee plantations in several southern provinces (upper part of the peninsula) resulted in more MCs being established. By 1999, there were 450 MCs in 33 zone offices and by 1998, there were 535 clinics and over 15,000 village malaria volunteers (VMVs) countrywide (AR, 1998). The number of MCs peaked in 1997, with 544 in operation.

From 1980 to 2000, incidence of malaria fell by 87% as the number of MCs continued to increase (Figure 3). The negative trend in malaria incidence began approximately three years after the expansion of MCs. Malaria mortality also declined by 87% over this twenty-year period (Figure 4). From 1988 to 1990, MCs examined 39.8% (*n* = 2,890,587) of all malaria slides—an unprecedented share for MCs (Figure 5). In the same three-year span, a record 54.8% (*n* = 505,372) of positive cases were detected at MCs (Figure 6). Although the proportion of positive cases detected at MCs initially declined after 1990, it has since remained relatively stable around 30–35%.

### 3.3. Malaria Clinics (MCs): 2000 to Present

The number of MCs declined from 544 (AR, 1997) in 1997 to 325 (AR, 2010) by 2009 because of the ASEAN (Association of Southeast Asian Nations)-wide financial crisis during 1997–1999. Nevertheless, the roles of MCs were expanded after 2009 due to increasing proportions of foreign nationals from neighboring countries being reported with malaria. Since 2009, long-lasting insecticidal nets (LLINs) have been provided free of cost to each unregistered migrant diagnosed with malaria at an MC. In response to reported artemisinin resistance in 2012, MCs commenced treatment with radical treatment of *P. falciparum* (Pf) through a directly-observed therapy (DOT) and followed up all Pf and *P. vivax* (Pv) positive malaria cases at day 28 of treatment [6]. This was possible through one dedicated microscopist and one staff member focused on information, education, and communication/behavior change communication (IEC/BCC) activities at the MC. In 53 MCs in seven border provinces with Cambodia, additional resources from the Global Fund to Fight AIDS, Tuberculosis and Malaria (GFATM) ensured the availability of two functioning microscopists and two microscopes. Further resources from GFATM in 2013 ensured employing workers at MCs for patient follow-up, foci investigation, and conducting mass blood surveys in ten provinces on the Myanmar border (AR, 2013).

Even with the closing down of some MCs since 2000, malaria incidence has continued its steady decline, dropping 95% (from 1.36 to 0.07 cases per 1000 population) between 2000 and 2019 (Figure 3). Malaria mortality has similarly declined, reaching a record low of 0.012 deaths per 100,000 population in 2018 (Figure 4). MCs in the current era examine a smaller proportion of total malaria slides (6.7% from 2015–2017) compared to MCs at the end of the 1990s (15.3% from 1997–1999) (Figure 5). While the number of total positive cases has decreased, the percentage of positives detected by MCs has changed little since the 1990s (Figure 6).

### 3.4. Role of MCs in Accelerating Malaria Elimination Since 2017

**Figure 7:**
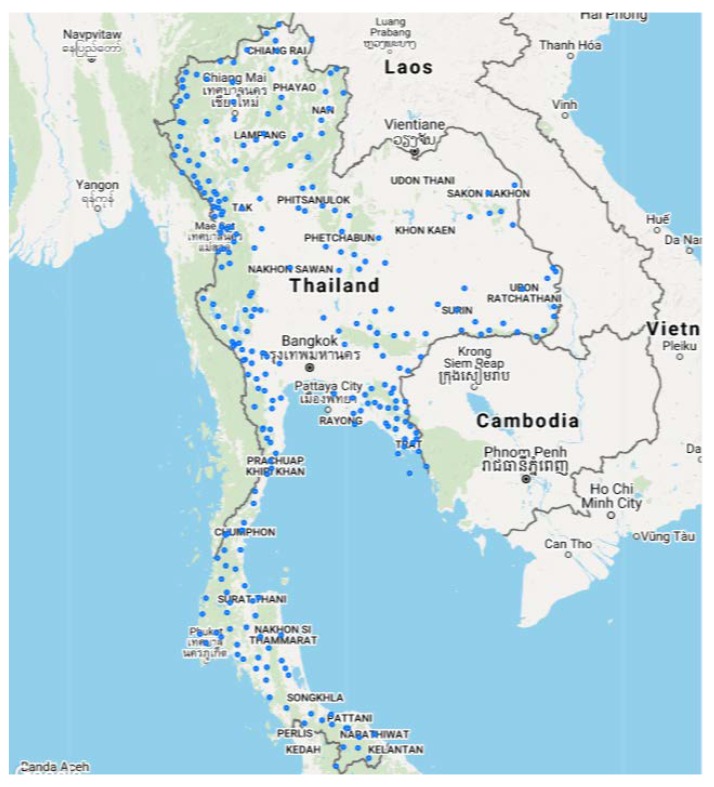
Distribution of MCs in Thailand as of 2018.

Since the launch of the malaria elimination strategy in Thailand in 2017, the number of MCs has remained fairly stable at 240 clinics nationwide (Figure 7). Geographically, most provinces have seen a reduction in annual malaria cases since 2017. While Mae Hong Son (19.3020° N, 97.9654° E) and Tak (16.8840° N, 99.1258° E) provinces (both bordering Myanmar) have had decreasing malaria, the proportion of these cases that are seen by MCs has been increasing (16 to 48% and 12 to 34.3%, respectively, from 2017 to the first half of 2019) (Table 1). However, the process of integrating the vertical malaria system into the general public health system necessitated redefining the implementing roles of all stakeholders.

#### 3.4.1. Redefining Implementing Roles for Malaria Elimination

The implementation unit of the elimination strategy is at the district level and involves redefining roles and responsibilities of all levels of the vertical malaria program, general public health services, local administration, and communities. This is summarized in Table 2. The role of MCs in this strategy is closely tied to the roles of the VBDU.

#### 3.4.2. Stratification by Foci and Case Classification

As the number of malaria cases in Thailand showed a steady decline at the start of this decade, malaria elimination was seen to be programmatically feasible. Stratification of malaria risk in Thailand was based on the WHO Malaria Elimination framework [17]. Thailand’s malaria elimination strategy 2017–2026 detailed elimination strategies based on case and vector surveillance data. Malaria foci and case classification allowed a more precise understanding of where and among which populations the source of transmission was occurring (see Table 3).

**Figure 8:**
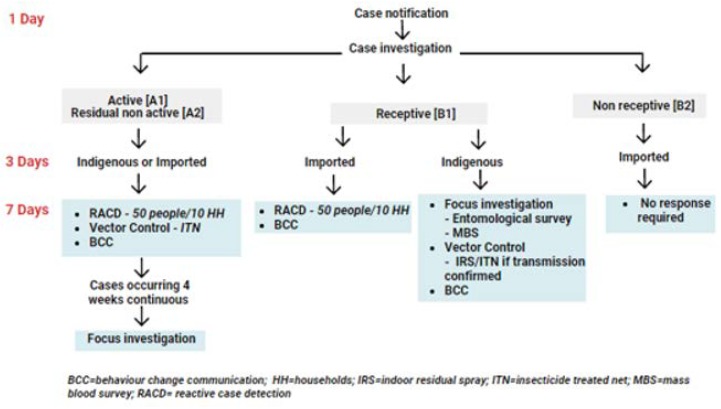
Thailand malaria elimination. 1-3-7 strategy: Case notification, investigation, and response. MBS = mass blood survey; BCC = behavior change communication.

Both foci and case classification have relevance to Thailand’s elimination strategy for surveillance and response, the “1-3-7” strategy, where case detection and notification is done within 1 day, case investigation is completed within 3 days, and an appropriate response to cut transmission within 7 days (as shown in Figure 8).

#### 3.4.3. MCs and Overall Contribution in Malaria Elimination on Malaria Testing and Positive Cases

**Figure 9:**
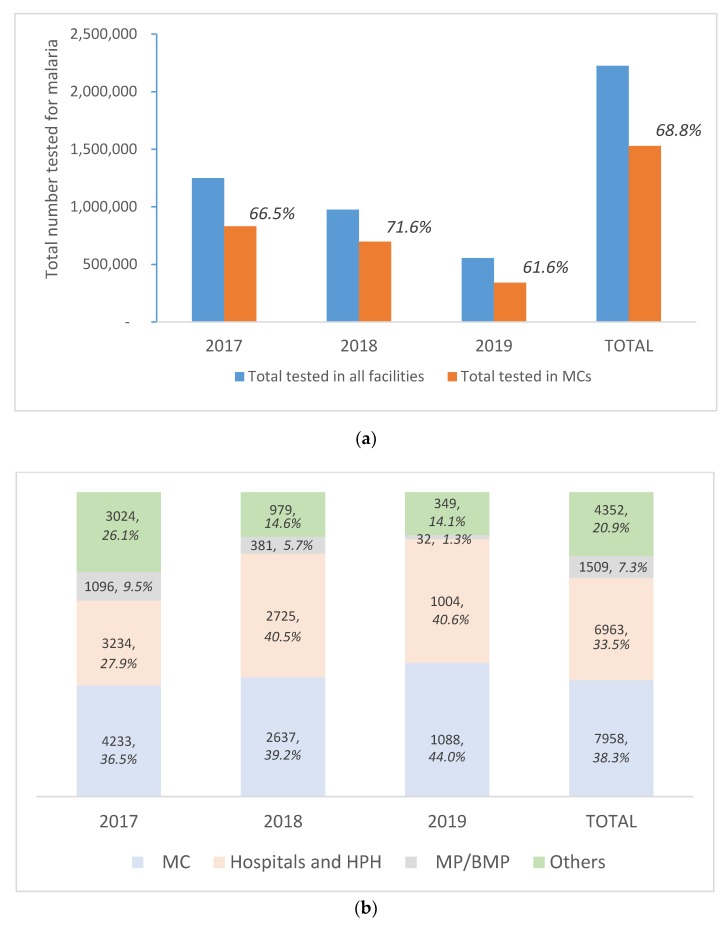
(**a**) Tested for malaria: Total all facilities and Malaria Clinics from 2017–2019 (June). (**b**) Malaria positive: Total by all health facilities from 2017–2019 (June). MP = malaria posts; BMP = border malaria posts.

MCs contributed an average of 66.7% to all testing for malaria in the country between 2017 and June 2019, with a 36.5% average yield of positive cases (Figure 9a,b). By type of facility, the highest proportion of positive cases detected came from the MCs (38.3%), with a trend of increasing yield annually over the same period from 2017 to June 2019 (positivity of 36.5%, 39.2%, and 44.0%, respectively).

#### 3.4.4. MC Contributions to PACD and RACD

In its malaria elimination strategy, the program intensified efforts to interrupt the transmission cycle through active and passive case detection. Passive case detection (PCD) involves malaria testing and treatment conducted in health facilities such as public and private hospitals, private clinics, MCs, and health promotion hospitals (HPH). In remote areas, malaria posts (MPs) or border malaria posts (BMPs) are established as a measure to improve access for malaria testing and treatment for all populations, regardless of legal status. Active case detection (ACD) is case detection and treatment conducted by health staff outside health facilities in the following situations:

1. Reactive case detection (RACD) is a case investigation survey initiated when an indigenous case is found in a transmission village or non-transmission village, but with the presence of a vector (A1/A2/B1). Blood is taken for microscopy from all members living in the patient’s house and all neighbors living around the index patient’s house, aiming for at least 50 blood samples or no less than 10 households within a 1-km radius.
2. Proactive case detection (PACD) is that blood is taken for microscopy but not prompted by an index case. It is done periodically, sometimes scheduled, among populations at-risk for malaria who live in a malaria transmission village (A1/A2) or those who have entered malaria-risk areas at night within the last two weeks.

Two other case finding activities that have historically been the task of the MC are the MMC and FSMC (vide supra). Mass blood survey (MBS) is conducted as part of foci investigation and to detect asymptomatic infected persons; it is taken by blood examination from everyone in the village or hamlet.

**Figure 10:**
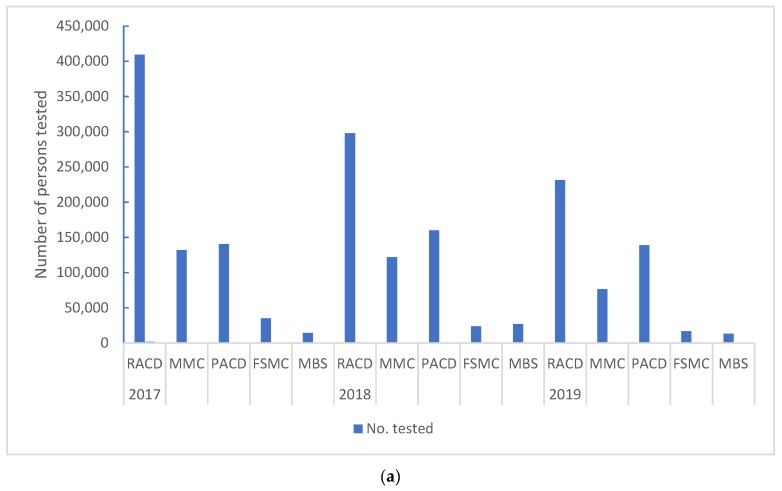

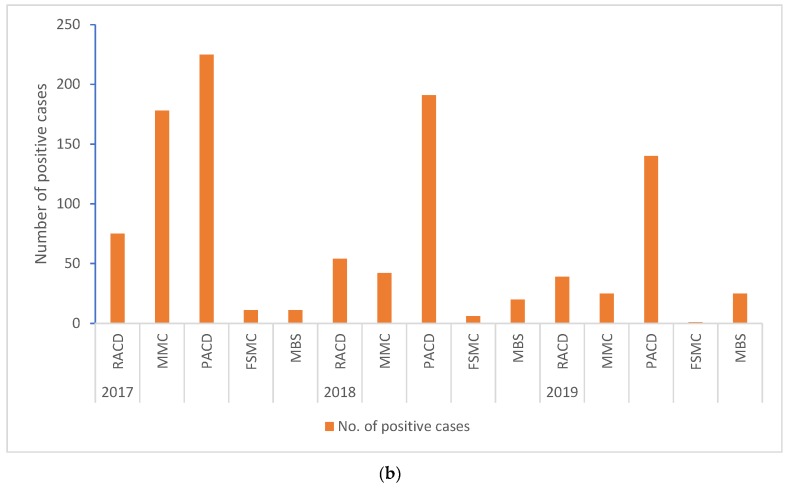
(**a**) Malaria Clinics: Number tested among different ACD approaches from 2017–2019 (June). (**b**) Malaria Clinics: Number positive among different ACD approaches from 2017–2019 (June).

As a PCD facility, MCs reported a slight decline in the number tested, number of positive cases, and positive rates over 2017 (99,784, 3691, 3.7%), 2018 (77,569, 2292, 3.0%), and first half of 2019 (32,491, 905, 2.8%). When analyzing the different ACD approaches undertaken by the MC over the period 2017–June 2019, different rates of testing were noted (Figure 10a). The average contribution was highest in RACD (50.6%), followed by PACD (24.6%), MMC (17.7%), FSMC (4.1%), and MBS (3.0%). All approaches were relatively constant over this period, with only PACD showing an increase (2017: 19.2%; 2018: 25.4%; and 2019: 29.1%).

Over the same period, the number of positive cases resulting from these ACD tests was notable (Figure 10b), with the highest average yield from PACD (55.6%), followed by MMC (19.9%), RACD (16.4%), MBS (6.5%), and FSMC (1.5%). Only RACD positive rates remained relatively constant over this period, with PACD showing an increase (2017: 45%; 2018: 61%; and 2019: 61%) and MBS (2017: 2.2%; 2018: 6.4%; and 2019: 10.9%). MMC yields saw a decline (2017: 35.0%; 2018: 13.4%; and 2019: 10.9%) and FSMC averaged below 2%.

#### 3.4.5. MCs and Trends in Foci and Case Classification

MCs’ contribution to malaria cases in the B1 and B2 (cleared foci) strata decreased from 52.3 to 38.0% and from 46.1 to 31.5%, respectively, over the same period (Table 4). MCs diagnosed a lower proportion of indigenous cases (68 to 50.3%) but a higher proportion of Bf (imported) cases (34.7 to 38.9%) (Table 5). They also diagnosed a smaller share of Thai nationals (7.9% drop from 2017) and a greater share of nationals from Myanmar (25.4% increase from 2017) (Table 6).

#### 3.4.6. MCs in Elimination: Relevance in Different Transmission Settings

In active foci (A1) areas between 2017 and 2019 (till June), 54% (*n* = 4277) of cases were detected in MCs compared to 54% (*n* = 2205) in residual non-active foci (A2) areas. Indigenous cases (A and Bx cases) accounted for 78% of total cases in A1 compared to 73% in A2. Of these, MCs in A1 detected 57% (*n* = 3483) and MCs in A2 59% (*n* = 1784) of indigenous cases (A and Bx cases), although the yearly trend was decreasing in both. Conversely, over the same period, imported cases (Bf) detected at MCs in both A1 (36%) and A2 (35%) foci showed a steady increasing trend over the same period. (Table 7).

In cleared but receptive foci (B1) areas between 2017 and 2019, 47% (*n* = 1231) of cases were detected in MCs compared to 40% (*n* = 579) in cleared and non-receptive foci (B2) areas. Indigenous cases accounted for 48% of total cases in B1 compared to 23% in B2. In both B1 and B2, reported indigenous cases on a yearly basis showed a decreasing trend. Moreover, over the same period, imported cases (Bf) detected at MCs in B1 (41%) and B2 (41%) showed a yearly fluctuating trend.

## 4. Discussion

**Figure 11:**
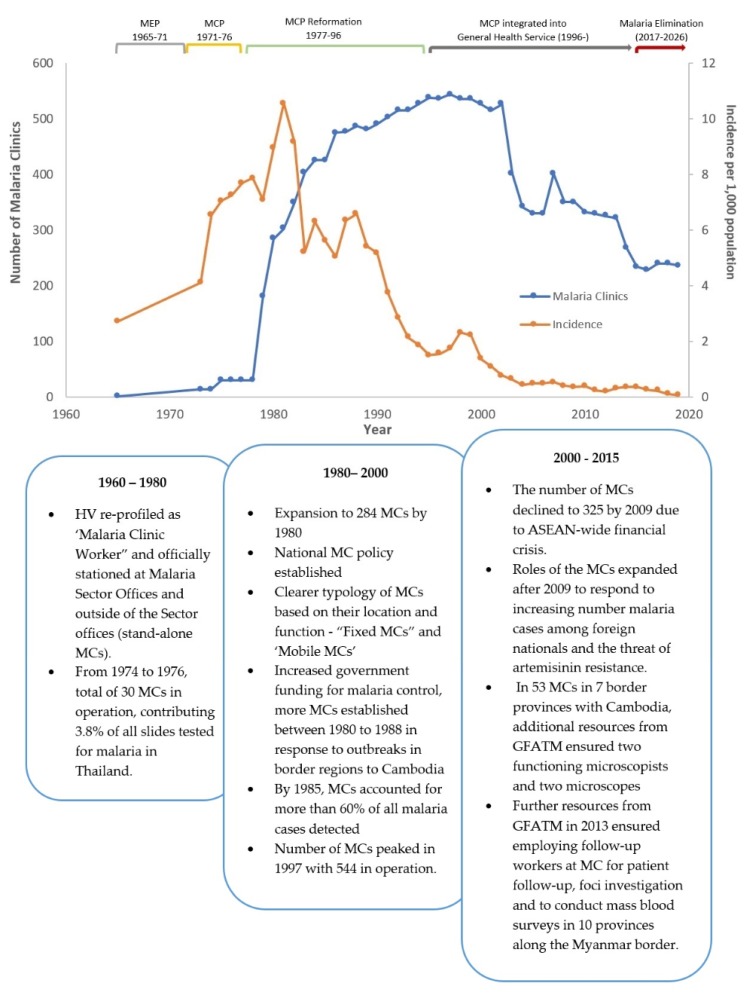
Overview of key evolution milestones of MCs in relation to phases of the malaria program in Thailand: 1960–2017. MEP = Malaria Eradication Program; MCP = Malaria Control Program; ASEAN = Association of Southeast Asian Nations; GFATM = Global Fund to Fight AIDS, Tuberculosis and Malaria.

The evolution, roles, and contribution of MCs since their establishment until 2015 is summarized in Figure 11.

The set-up of the MC in Thailand is unique. There are a few parallels with other countries in the region of similar extensions like MC that arise from the malaria program vertical system. Although the authors did not conduct an in-depth literature review of successful malaria programs where MC-like structures have been employed, the use of MCs in three highly successful malaria elimination programs in the region was studied. These MC-like initiatives were part of the formal ministry structure, thus were government funded as opposed to project or donor funded; therefore, they were more sustainable over time. The use of subsector malaria offices in remote parts of Sabah, Malaysia [18], mobile malaria clinics in Sri Lanka [19,20] and malaria control consultation and service posts (MCCSPs) along the border areas of Yunnan province, P.R. China [21], showed that despite specific differences and contextual circumstances in their initial development, there were commonalities in all three countries.

Firstly, the primary objective was to hasten and improve access to diagnosis and treatment for malaria patients with a minimum delay after the onset of the illness. Although disaggregation of data by facility and robust analysis was not possible in the initial years of operation, the MC service in Thailand significantly improved patient turn-around time to 30–40 min between diagnosis and treatment as compared to a month. Patient satisfaction with this service proved to be an important impetus that not just established the popularity of MCs, but also led the program managers to further expand its utility. By being responsive to the changing epidemiology and the need to reach different at-risk populations, including outbreaks, the Thai program began diversifying MC service delivery channels from “fixed MCs” within existing facilities and stand-alone MCs to MMCs and FSMCs. Along the geographical expansion, the contribution of MCs to blood slides tested increased 10-fold (39.8% of all malaria slides from 1988 to 1990) compared to the previous decade, with a record 54.8% positivity rate of those tested at MCs. Although the proportion of positive cases detected at MCs initially declined after 1990, it has since remained relatively stable around 30–35%.

Secondly, there was government investment in not just the initial infrastructure (building, vehicle, equipment, etc.), but also the employment, training, and on-going supervision of microscopists, field officers, laborers and occasionally a Public Health Inspector at MCs as in Sri Lanka. The Thai program proved that even though domestic financial constraints in the early 2000s necessitated the closing of a few MCs, it was still possible to focus on current challenges: The increasing number of unregistered migrants diagnosed with malaria and the emergence of artemisinin and multi-drug resistance. MCs, especially in areas bordering Myanmar and Cambodia, diversified by providing free long-lasting insecticidal nets (LLINs) for unregistered migrants diagnosed with malaria and commencing radical treatment for Pf with supervised treatment until day 3, and a follow-up on days 7, 28, and 42. Further investments from donor agencies allowed the program to employ additional human resources (microscopist, additional microscopes, and IEC/BCC staff and workers) to follow up with patients, as well as to conduct foci investigations and mass blood surveys.

**Figure 12:**
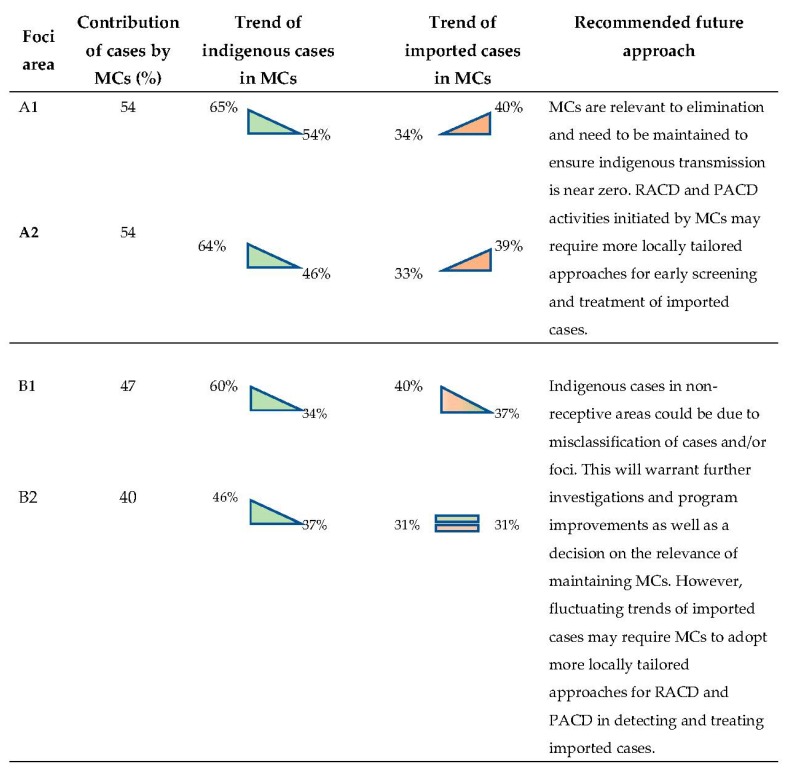
Current trends: 2017–June 2019. MCs’ role in malaria elimination and recommended approaches.

Thirdly, in areas where malaria burden declined to very low levels as with elimination settings, MCs focused more on mobile or outreach activities to screen the populations of districts with high vulnerability and/or receptivity for malaria and to maximize case detection through RACD or PACD. This allowed for a higher yield in case finding and, through prompt and effective treatment, reduced the parasite reservoir and the possibility of further transmission. The Thai program, in its nascent elimination phase, should learn where and how MCs can be best utilized in ACD activities from the three MC-like examples. In leading up to the WHO certification of elimination, these approaches will strengthen the evidence that there is no indigenous transmission in areas that were screened actively and periodically through the outreach of the MCs. A proposed framework for this is outlined in Figure 12. This figure looks at cases in the four different foci areas, specifically the proportion of these cases that were seen by MCs and the percentage of cases within MCs that were indigenous or imported. Based on the trends in these numbers, we recommend an approach to malaria elimination tailored to each foci area.

The authors acknowledge certain limitations in this research. The authors relied largely on secondary data from both published articles and unpublished reports and data, annual reports (ARs) obtained from the DVBD and Department of Communicable Disease Control, Ministry of Public Health, Thailand from 1975 to 2005. The years 1965–1974 were omitted in this research because of missing data on MCs in some years. Personal records of the senior staff members that were interviewed, although extremely valuable, could not be validated. Due to limited resources for this research, the authors could not conduct interviews with either retired or current MC staff. Although we analyzed and presented the main outcomes of the 1-3-7 on trends of transmission, such as case profile and foci, the actual performance of the MCs against the national program indicators for monitoring the performance of 1-3-7 was not assessed. Case notification, investigation, and response rates specifically disaggregated by MCs could not be performed due to technical issues with the web-based surveillance platform.

Another important area for further research would be to analyze in detail the human resource situation, especially at the district and lower levels (elimination implementation unit). A detailed profiling of staff (staffing numbers, anticipated retirement age, capacity gaps, workload required to meet elimination targets, etc.) within the vertical malaria network of VBDU and MCs will be critical. This would require the use of qualitative tools to appraise and the exploration of future replacement strategies at the district level critical about the roles and resources of the CDCU, district health office (DHO) local administration organizations, health promotion hospitals, private health facilities, and civil society organizations. This analysis should be performed to be in line with the positioning of malaria elimination as a part of the general health services and with the 20-year Thailand National Health Strategic Framework (2017–2036).

## 5. Conclusions

Malaria clinics have served communities in Thailand for almost six decades and are still playing a critical role in providing early diagnosis and effective treatment of malaria. Between 2017 and June 2019, during the malaria elimination phase, MCs continued to test an average of 67% of all persons tested for malaria and confirmed 38% of all positive cases detected in the country. However, both the testing and positive rates are on a gradual decline as the overall burden of malaria declines annually, which may reflect decreasing transmission intensity. Since 2000, several MCs in areas with zero reported malaria cases and absence of mosquito vectors have been closed. Most experienced field MC staff have retired. Although the number of MCs in the last three years has been stable (*n* = 240), the attrition of MC staff, especially microscopists, poses a real challenge to the longevity of MCs in the absence of a human resource plan to support the elimination phase.

As Thailand’s malaria program becomes increasingly decentralized and integrated into general health services, it will be important to maintain the technical and programmatic capacity to manage core malaria services, including analysis and decision-making, at the sub-national level. Although the CDCUs are initially expected to take over functions of the MCs with regard to the case and foci management in areas where malaria transmission is very low (B1 areas), it will be necessary to identify and support capacity gaps and needs during this period of transition from a strictly vertical program to an integrated and decentralized program, while ensuring that the DVBD maintains its necessary technical and advisory role for malaria control and elimination within the MoPH.

## Figures and Tables

**Table 1:** Malaria cases by province and health facility (MC vs. all other facilities) in Thailand between 2017 and June 2019.

	2017			2018			2019		
Province	Cases	MCs	% Represented by MCs	Cases	MCs	% Represented by MCs	Cases	MCs	% Represented by MCs
Bangkok	25	0	0.00	18	0	0.00	13	0	0.00%
Chachoengsao	63	10	15.90	47	4	8.50	11	1	9.10
Chanthaburi	158	100	63.30	93	49	52.70	8	5	62.50
Chiang Mai	49	9	18.40	16	1	6.30	6	1	16.70
Chiang Rai	68	9	13.20	10	1	10.00	6	2	33.30
Kanchanaburi	245	90	36.70	317	158	49.80	214	100	46.70
Mae Hong Son	394	63	16.00	342	106	31.00	100	48	48.00
Narathiwat	347	108	31.10	74	22	29.70	44	2	4.50
Pattani	52	37	71.20	19	10	52.60	2	1	50.00
Phetchaburi	96	33	34.40	64	34	53.10	128	46	35.90
Prachinburi	156	58	37.20	39	17	43.60	1	0	0.00
Prachup Khiri Khan	83	39	47.00	84	30	35.70	108	49	45.40
Ranong	124	29	23.40	42	17	40.50	38	16	42.10
Ratchaburi	106	20	18.90	146	28	19.20	139	16	11.50
Si Sa Ket	952	345	36.20	903	260	28.80	125	40	32.00
Songkhla	246	158	64.20	88	43	48.90	83	62	74.70
Tak	4099	493	12.00	1880	534	28.40	794	272	34.30
Trat	70	49	70.00	48	32	66.70	19	10	52.60
Ubon Ratchathani	297	87	29.30	527	177	33.60	89	19	21.30
Yala	3429	2316	67.50	1541	1005	65.20	694	380	54.80
TOTAL	11,059	4053	35.30	6298	2528	35.22	2622	1070	33.77

Cases diagnosed in all other health facilities, including public and private hospitals, health promoting hospitals, and malaria posts.

**Table 2:** Malaria elimination in Thailand: Summary of key implementation roles at the district and lower administrative levels.

	Main Accountability	Contributory	Supervision by
**Vertical Malaria Network**			
VBDU	Surveillance and risk stratification Case and foci investigation, classification ACD–RACD and PACD IRS/LLIN/ITN implementation	Support for passive case detection in HPH, and MC	VBDC
Malaria Clinics	All activities related to ACD–RACD, PACD, mobile clinics	Support VBDU and CDCU	VBDC
**Hospital and Curative services**			
DH	Passive case detection Case notification Case management	UHC link to HPHs	VBDC and DHO
HPH	Passive case detection Case notification Case management—uncomplicated malaria	ACD–RACD Training and support to HVs	DH and VBDU and DHO
**District/Subdistrict Administration**			
CDCU	Rapid response team for all communicable diseases (exist at provincial, district, and sub-district levels)	Jointly conducts all main responsibilities of VBDU and MC Assess vulnerability and receptivity risks	DHO
LAO and municipality	Source reduction strategies Community mobilization—behavior change communication	Utilization of mosquito nets	Technical support from VBDU and DHO
**Village**			
Health volunteers	Referral of fever cases Utilization of bed nets Patient treatment follow up	Source Reduction	HPH and VBDU

ACD = active case detection; CDCU = communicable disease control unit; DH = district hospital; DHO = district health office; HPH = health-promoting hospital; HV = health volunteer; IRS = indoor residual spraying; ITN = insecticide-treated net; LAO = local administrative organization; LLIN = long-lasting insecticide-treated net; PACD = proactive case detection; RACD = reactive case detection; UHC = universal health coverage; VBDC = vector borne disease control center; VBDU = vector borne disease control unit.

**Table 3:** Thailand malaria elimination program: Foci and case classification system.

Variable	Definition
**Foci classification**	
A1	Active foci
A2	Residual non-active foci
B1	Cleared foci but receptive *
B2	Cleared foci but not receptive *
**Case classification**	
A	Indigenous case (acquired in village)
Bx, By, Bz, Bo	Imported case Bx: Outside village; By: Sub-district, Bz: District, Bo: Province
Bf	Imported case (outside country)
F	Unclassified case

* Receptivity defined as presence/absence of *Anopheles* species.

**Table 4:** Malaria cases by foci classification in Thailand between 2017 and June 2019.

Variable	2017		2018		January–June 2019	
Foci	All Cases	Cases at MCs (%)	All Cases	Cases at MCs (%)	All Cases	Cases at MCs (%)
A1	3031	1483 (48.9)	3437	1713 (49.1)	1686	782 (46.4)
A2	3295	1768 (53.7)	795	230 (28.9)	356	144 (40.4)
B1	1150	601 (52.3)	990	438 (44.2)	313	119 (38.0)
B2	590	272 (46.1)	695	252 (36.3)	149	47 (31.5)
Unknown	3520	104 (3.0)	757	5 (0.7)	215	4 (1.9)

A1: Active foci; A2: Residual non-active foci; B1: Cleared but receptive foci; B2: Cleared and non-receptive foci; Unknown: Foci with classification undetermined.

**Table 5:** Malaria cases by case classification in Thailand between 2017 and June 2019.

Variable	2017		2018		January–June 2019	
Case Classification	All Cases	Cases at MCs (%)	All Cases	Cases at MCs (%)	All Cases	Cases at MCs (%)
A	3883	2641 (68.0)	2343	1318 (56.3)	1186	597 (50.3)
Bx, By, Bz, Bo	1952	977 (50.1)	1771	725 (40.9)	426	190 (44.6)
Bf	1028	357 (34.7)	965	362 (37.5)	633	246 (38.9)
F	251	70 (27.9)	200	82 (41.0)	32	15 (46.9)
Unknown	4472	184 (4.1)	1443	149 (10.3)	439	48 (10.9)

A: Indigenous case (acquired in village); Bx: Imported case (acquired outside village, but within sub-district); By: Imported case (acquired outside sub-district, but within district); Bz: Imported case (acquired outside district, but within province); Bo: Imported case (acquired outside province, but within country); Bf: Imported case (acquired outside country); F: Unclassified case.

**Table 6:** Malaria cases by nationality in Thailand between 2017 and June 2019.

Variable	2017		2018		January–June 2019	
Nationality	All Cases	Cases at MCs (%)	All Cases	Cases at MCs (%)	All Cases	Cases at MCs (%)
Thai	7317	3741 (51.1)	4874	2104 (43.2)	1853	800 (43.2)
Other	4172	488 (11.7)	1770	533 (30.1)	859	150 (17.5)
Cambodia	109	42 (38.5)	75	36 (48.0)	15	7 (46.7)
Myanmar	3819	381 (10.0)	1443	422 (29.2)	692	245 (35.4)
Laos	36	4 (11.1)	25	8 (32.0)	4	2 (50.0)
Other	208	61 (29.3)	227	67 (29.5)	148	42 (28.4)

**Table 7:** MCs in elimination: Relevance in different transmission foci: 2017–2019 (June).

	2017–2019								2017	2018	2019	2017	2018	2019
Foci area	No. and % Cases in MCs		No. and % A and Bx Cases Nationwide		No and % of A and Bx Cases in MCs		No and % of Bf Cases in MCs		Proportion of A and Bx Cases Detected in MCs			Proportion of Bf Cases Detected in MCs		
	n	%	n	%	n	%	n	%	%	%	%	%	%	%
**A1**	4277	54	6064	78	3483	57 *	416	36 *	65 *	55 *	54 *	34 *	36 *	40 *
**A2**	2205	54	3002	73	1784	59 *	240	35 *	64 *	46	40 *	33 *	34 *	39 *
**B1**	1231	47	1225	48	646	51 *	307	41 *	60 *	47	34 *	40 *	44 *	37 *
**B2**	579	40	331	23	140	42 *	81	41 *	46	41 *	37	31 *	34 *	31 *

* Statistically significant difference, *p*-values, *p* < 0.05.
